# Supplementary material for: VvPL15 Is the Core Member of the Pectate Lyase Gene Family Involved in Grape Berries Ripening and Softening
Source: Int J Mol Sci. 2023 May 26;24(11):9318. doi: 10.3390/ijms24119318 (PMC10253464; doi:10.3390/ijms24119318)
Supplement: Supplementary file 1 [file ijms-24-09318-s001.zip › ijms-2290210-supplementary.pdf]

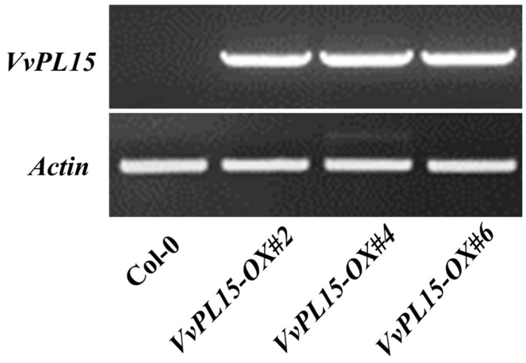

Figure S1. Identification of the *VvPL15-OX* Arabidopsis seedlings at the DNA level.

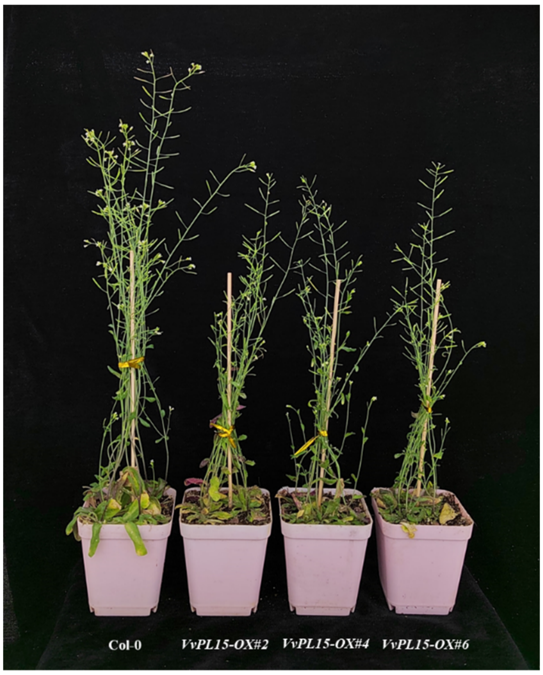

Figure S2. The phenotype of *VvPL15* overexpression.

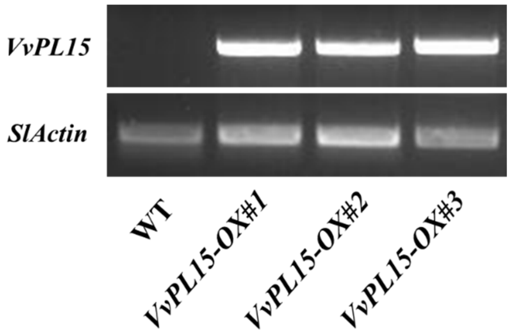

Figure S3. Identification of the *VvPL15-OX* tomato at the DNA level.

Table S1. Number of cis-elements occurrence in *VvPL* gene promoters.

Gene name	Genome v2.1	TGA-element	P-box	ERE	ABRE	MYB	TCA-element	CGTCA-motif	W-box	LTR	MBS
VvPL1	VIT_01s0026g01670	0	1	3	4	8	1	2	2	3	2
VvPL2	VIT_01s0026g01680	1	1	4	5	6	1	0	0	0	1
VvPL3	VIT_01s0137g00240	1	1	0	9	5	1	6	1	0	2
VvPL4	VIT_05s0051g00590	0	1	4	9	6	0	2	2	1	0
VvPL5	VIT_07s0005g05520	0	0	1	11	9	1	2	0	0	0
VvPL6	VIT_08s0007g04820	0	0	3	3	3	1	0	1	0	0
VvPL7	VIT_08s0040g02740	0	0	3	0	3	1	2	1	0	0
VvPL8	VIT_10s0116g01160	0	0	5	1	3	0	0	0	0	0
VvPL9	VIT_13s0019g04900	2	2	7	0	0	1	0	1	0	0
VvPL10	VIT_13s0019g04910	0	1	8	0	2	0	0	1	0	1
VvPL11	VIT_14s0108g00030	0	0	0	2	4	0	0	0	0	0
VvPL12	VIT_14s0219g00230	0	0	2	3	7	0	6	1	1	1
VvPL13	VIT_16s0039g00260	0	1	2	2	6	1	0	0	1	1
VvPL14	VIT_17s0000g05740	0	1	1	4	9	0	0	1	0	1
VvPL15	VIT_17s0000g09810	2	0	1	0	2	4	4	2	0	0
VvPL16	VIT_19s0014g00510	0	0	2	0	7	0	0	1	3	0

Table S2. Primers used in this study.

Gene name	Sequence (5' to 3')
VvActin-F (qRT-PCR)	GAGATTCCGTTGTCCAGAAGTC
VvActin-R (qRT-PCR)	CAATGTTGCCATAGAGGTCCTT
VvPL1-F (qRT-PCR)	TTCACAACGCAAAAGGCTAGC
VvPL1-R (qRT-PCR)	GCATGGCGTAATGTTCCCTTC
VvPL2-F (qRT-PCR)	AATCCTAAGCCTGGAACCTTACG
VvPL2-R (qRT-PCR)	GTTTTGTCACTGGTCACCATCAG

VvPL3-F (qRT-PCR)	GTATGAAGGTGAAGACAAGTGC
VvPL3-R (qRT-PCR)	TCTTCGTTTCTTCTTTACCCCA
VvPL4-F (qRT-PCR)	GTCTAGAGAGAGAGCTTTCGAC
VvPL4-R (qRT-PCR)	CAGCCATTGATGAGTTGTTTGA
VvPL5-F (qRT-PCR)	CCTTTATGGATCGTGTTCAAGC
VvPL5-R (qRT-PCR)	GATAAACTGGATGGTGATGCAC
VvPL6-F (qRT-PCR)	GTCACAGCGATTCGTATGTGC
VvPL6-R (qRT-PCR)	TGACCACGTGTATATAGCCGC
VvPL7-F (qRT-PCR)	TGGTGATAGATGTATTCGGGTG
VvPL7-R (qRT-PCR)	CAGCTGCCTTCTCTGTAAGATA
VvPL8-F (qRT-PCR)	CCACGTGGTGAATAACGATTAC
VvPL8-R (qRT-PCR)	CTCCATCTTTTGCATCCATACG
VvPL9-F (qRT-PCR)	CCACGTGGTGAATAACGATTAC
VvPL9-R (qRT-PCR)	CTCCATCTTTTGCATCCATACG
VvPL10-F (qRT-PCR)	CAGAGCCACTCTCAACCTCAAT
VvPL10-R (qRT-PCR)	ACAGAGAGCACTTGTCTTCTCG
VvPL11-F (qRT-PCR)	TCCAGGGCGATAAGATCATGC
VvPL11-R (qRT-PCR)	GTTGACAACATGGACGAAGCC
VvPL12-F (qRT-PCR)	GTGCTGACCCTACCATCAACA
VvPL12-R (qRT-PCR)	ATGCCTCCACTCACTTTCTGG
VvPL13-F (qRT-PCR)	GTTGCATCCTCACAAATTTGGA
VvPL13-R (qRT-PCR)	CTGAACCAGTTGTTTGAGATGG
VvPL14-F (qRT-PCR)	AATTTTCAGGACTATGCATGGC
VvPL14-R (qRT-PCR)	CAAGCACACAATCAGCTAGTTT
VvPL15-F (qRT-PCR)	TGAACTCCTTCAAGACCATTGA
VvPL15-R (qRT-PCR)	ACCGTCTGATATCGTCCTAAAC
VvPL16-F (qRT-PCR)	ACGGACTCATTGATGCAGTGT
VvPL16-R (qRT-PCR)	TCATCACTATGCCCCATGAGC
VvPL15-F (pRI)	ttgatacatatgcccgtcgacATGGCTAGCTCTTCACTTTCCCT
VvPL15-R (pRI)	agagttgttgattcagaattcTCAGCATCGAGAACCTTTCTTGC
VvPL15-F (TRV)	gtgagtaaggttaccgaattcATGCAGTCATCCAAGATGAGCC
VvPL15-R (TRV)	cgtgagctcggtaccggatccAGATATGGCTCCCACCAAAGATG
AtActin2-F	ATCAGCCGTTTTGAATCTCC
AtActin2-R	CAATCTAACTTCAACAGTTC
